# Reviews and Book Notices

**Published:** 1881-11

**Authors:** 


### Reviews and Book Notices.

ARTICLE XIII.—LECTURES ON THE DIAGNOSIS AND TREATMENT OF DISEASES OF THE CHEST, THROAT AND NASAL CAVITIES. By E. FLETCHER INGALS, A.M., M.D., Lecturer on Diseases of the Chest and Physical Diagnosis, and on Laryngology, in the Post-graduate Course, Rush Medical College; Clinical Professor of Diseases of the Throat and Chest, Woman's Medical College; Physician and Surgeon for Diseases of the Throat and Chest, Central Free Dispensary, Chicago. With one hundred and thirty-five illustrations, pp. 437. Wm. Wood & Co., New York, 1881.

This forms a valuable aid, both to the student and practitioner, in the study of its subject. It is clear and concise in style, systematic and thorough in the consideration of each detail. The author presents the results of wide personal experience in this specialty, and exhaustive study of the literature already before the profession upon the subject.

It comprises thirty-three lectures, the first of which is devoted to an elaborate study of the regional anatomy of the thorax. Lectures II to IX contain the methods of examination of the chest in health and disease; those on inspection (and under this head are found many useful hints to the student), palpation, mensuration, succussion, percussion and auscultation. The analysis of the two latter we think particularly fine. The elements of the sounds obtained by percussion in health and disease are described as to intensity, pitch, quality and duration, in every variety of condition, from clear vesicular resonance to absolute flatness, and precise rules prescribed to the student in percussion. In auscultation the same elements are considered, with the addition of rhythm, through each modification of the respiratory act, from the soft breeze of the vesicular murmur to the loud blowing of the trachea.

Special care is enjoined throughout upon the student to become familiar with the sounds of the healthy chest; "to form an ideal from which no great variation can occur without indicating disease." Lectures IX to XV comprise the Diagnosis and Treatment of Pulmonary Diseases, and we find that the thorough preparation in the preceding lectures leads up to the grouping of the symptoms and signs of each disease in clear and lucid style. The differential diagnosis of each disease receives great care, and tables are appended wherein the essential difference in the signs of diseases liable to be confounded with each other are distinctly shown under each method of examination.

Lectures XV to XX comprise the Anatomy, Relations and Physiology of the Heart and Aorta. The methods of cardiac exploration each receive careful detail, with the information obtained by it; and here we notice, under the head of inspection, a table illustrating the significance of alterations in the situ ation of the apex beat, which conveys a good deal of information in small space.

The sounds of the healthy heart and their modifications receive due consideration under auscultation. Two chapters are given to abnormal sounds of the heart and their differential diagnosis. The cuts illustrating this subject are clear and plain. Lectures XX, XXI and XXII present a complete exposition of cardiac diseases and the diseases of the aorta, and here, under each head, the confrontation tables contribute to its clearness.

The remainder of the book is devoted to the fauces, larynx and nasal passages. Four chapters on laryngoscypy and rhinoscopy, description, difficulties of and rules for, and the anatomy and physiology of the larynx and post-nasal space, followed by the diagnosis and treatment of all the diseases of the throat and nasal cavities known to present science.

The chapters on the larynx are especially well illustrated. The volume is well furnished with table of contents and index, and a handy list of formulæ is appended, in which are found the remedies used by the author, with others compiled from various sources.

When these lectures were delivered the subject of treatment was not mentioned, but in order to enhance their value in this form, the author has appended to the consideration of the diagnosis of each disease an outline of the treatment found by him most satisfactory, without mention of many different methods recommended by others.

The special points to be commended in this work are, thoroughness of consideration, method of arrangement for study, and availability of the contents for reference. W. C. D.

---

THE first meeting of the State Microscopical Society of Illinois for the present season was held at the rooms of the society in the Academy of Sciences, Friday evening, Oct. 14, the President, Dr. Lester Curtis, in the chair.

After the transaction of routine business, Mr. Stuart described the microscopical structure of some vegetable drugs. The subject is not suitable for abstraction, and requires illustrations to be useful.

His paper was followed by one by Dr. Curtis, describing a new stand made for him by Bulloch. This stand presented some novel features. Among the most striking was a mechanical stage of extreme thinness, admitting light at an angle of 160°. The movements were effected by a double pinion above the stage, an arrangement pronounced by those familiar with the operation of the contrivance as exceedingly useful and convenient.

The stand excited considerable attention, as did also a right angled camera lucida of German manufacture which was adapted to it; the superiority of which over the ordinary form was so marked as to be unmistakable on trying it, even under the disadvantages of a crowded room and constant jar. After a discussion of the papers, the meeting adjourned.

E. B. STUART, *Secretary pro tem.*

